# Optimisation of Thermal Processes with Plasma Nitriding on Vanadis 4 High Speed Steel

**DOI:** 10.3390/ma15030906

**Published:** 2022-01-25

**Authors:** Florentino Alvarez-Antolin, Alejandro Gonzalez-Pociño, Alberto Cofiño-Villar, Carlos Hugo Alvarez-Perez

**Affiliations:** Department of Material Science and Metallurgical Engineering, University of Oviedo, Independencia 13, 33004 Oviedo, Spain; UO229780@uniovi.es (A.C.-V.); alvarezhugo@uniovi.es (C.H.A.-P.)

**Keywords:** tool steel, Vanadis 4, wear, nitriding, retained austenite, destabilization of austenite, secondary carbides, VC

## Abstract

Vanadis 4 steel is a tool steel processed by powder metallurgy (PM). Its main alloying elements are Cr, V and Mo. Through the application of a design of experiments with six factors and eight experiments, the parameters of the process related to the thermal treatment of these steels are deliberately varied. Those thermal parameters related to the destabilisation of austenite were analysed: the cooling method in quenching, tempering and the application of an ionic nitriding treatment. Through XRD, the percentage and types of precipitated crystalline phases were determined, and, through SEM-EDX, the microstructure was revealed. At the same time, through a pin-on-disc test, those factors with a significant influence on resistance to wear were determined. It has been concluded that, in order to increase resistance to wear, treatments of destabilisation of the austenite at 900 °C with oil quenching, tempering at 550 °C for 4 h and a subsequent treatment of plasma nitriding would be very favourable. This tempering favours a second destabilisation of the austenite and its transformation into martensite, as well as the joint precipitation of type M_7_C_3_ and MC carbides. The thickness of the nitrided layer exceeds 100 microns and generates a fully adhesive wear mechanism.

## 1. Introduction

Vanadis 4 is a tool-making steel processed by powder metallurgy (PM) and marketed by the UDDEHOLM company (Hagfors, Sweden). These steels are used in the manufacturing of equipment used in the cold forming of materials. In these steels, a specific set of properties is required, such as great hardness and resistance to wear, so their carbon content is high. Vanadis 4, as a consequence of its powder metallurgical origin, presents optimal toughness [1]. Steels manufactured by conventional casting show a high dendritic segregation and a large eutectic carbide network. This leads to low toughness. In order to improve its toughness, it is necessary to carry out a homogenization annealing and a hot forging process so as to fragment and disperse this carbide network. Following this, these steels should be subjected to annealing to facilitate their machining. Through quenching and tempering, the required properties are obtained by the final user [2,3]. The process of forming by powder metallurgy (PM) allows for the elimination of dendritic segregation. It also avoids the hot forming process and the obtaining of homogenous properties in the entire piece [4]. Vanadis 4 steel contains a high percentage of C and alloyed elements (mainly Cr, V, Mo) so that the temperature for transformation of austenite into martensite during quenching is very low. Therefore, a high percentage of austenite could remain retained after quenching [4]. This austenite could reduce resistance to wear and reduce the service life of this steel [1]. Because of this, sub-zero treatments, which allow the elimination of retained austenite, are common [5,6,7,8,9]. The microstructure of this steel presents two types of carbides: MC, mainly associated with vanadium, and M_7_C_3_, formed mainly by chromium. The VC has a high level of hardness, 2800 HV, and high fusion temperature, 2830 °C. This carbide is very efficient in the inhibition of the growth of grains during the austenitising treatment before quenching [1]. This carbide has a crystalline network similar to that of sodium chloride. That is to say, in the vertices and in the centre of faces of a cube is where V is situated. C is situated in the centre of the edges and in the centre of the cube. Cr_7_C_3_ presents as a complex hexagonal cell formed of 80 atoms (56 of Cr and 24 of C). Its hardness reaches 1600 HV and its fusion temperature is 1765 °C. The Cr could also be substituted by Fe, forming mixed carbides of the type M_7_C_3_, also called K_2_ carbides [10]. As a result of destabilisation of the austenite, at temperatures of around 1000 to 1100 °C, secondary carbides rich in Cr are precipitated, mainly of the type M_7_C_3_. To achieve this, times of permanence at the austenitising temperature need to be above 4 h [11]. Destabilisation of the austenite increases the Ms temperature, thus decreasing the percentage of retained austenite [12,13]. During tempering, a secondary hardening could be produced due to the precipitation of carbides of the types M_7_C_3_ and M_23_C_6_, associated with Cr [9,14,15]. At low tempering temperatures, cementite carbides precipitate. However, at temperatures higher than 500 °C, a redissolution of these cementite carbides is produced because of the decomposition of atoms that make up these carbides (Fe and C). Later, the diffusion of atoms of Cr is produced, dissolved in a substitute solid solution, as well as the precipitation of type M_23_C_6_ and M_7_C_3_ carbides of nanometric size. This precipitation produces a structural hardening of the ferrite. However, if the tempering is prolonged, the coalescence of these carbides occurs, which would produce a smaller increase in hardness [16]. The V diffuses at a lower speed in the ferrite than in the Cr. Therefore, in order to produce the precipitation of VC during tempering, higher temperatures are needed [17]. On the other hand, double or triple tempering allows the transformation of a large part of the retained austenite into martensite [18]. Tempering favours a second destabilisation of the austenite and its transformation into martensite [19]. The transformation of retained austenite commences with tempering at 470 °C [9]. These types of steels could be subjected to a nitriding treatment. The tempered martensite favours the diffusion of N so that thermo-chemical nitriding treatments produce a superficial hardness due to the formation of sub-nitrides in the matrix of the tempered martensite. This favours an increase in resistance to wear. However, this treatment could negatively affect the resistance to fracture of the alloy [20]. The most recommended nitriding method for application on steel tools is plasma nitriding. This method favours the diffusion of N throughout the steel since it eliminates the possible presence of stable chrome oxides from its surface [21]. In the first stage of nitriding, nanoprecipitates of the type CrN are formed, coherent with the matrix. In a second stage, these precipitates would thicken, thus losing coherence with the matrix [21]. Nanoprecipitates of three to four atoms of thickness and various nm of longitude with VN coherent with the matrix are also formed. These precipitates present a much slower thickening than those mentioned previously [22,23]. The carbides present in the microstructure also absorb a certain quantity of N [21]. Nitriding is most effective when a certain tetragonality is maintained in the tempered martensite. In a cubic ferrite, the formation of Fe_4_N, which is brittle, is more favourable. All these circumstances justify an analysis that includes nitriding treatment and its relationship with thermal processing prior to said nitriding.

The aim of this paper is to study the effect of different process variables related to thermal treatments that may condition the resistance to wear of Vanadis 4 steel. In particular, this study analyses different parameters for the destabilisation of the austenite, different quench cooling media, different tempering parameters and the additional effect of a plasma nitriding treatment. The experimental methodology followed was that of a design of experiments with six factors and eight experiments [24]. The industrial objective is that tooling and die manufacturers can use the most appropriate industrial thermal treatment to optimise the in-service behaviour of these steels, thus avoiding having to use sub-zero treatments. Sub-zero quenching treatments are less common in the industry due to their technical complexity and because of the limiting condition of the size of the pieces.

## 2. Materials and Methods

Table 1 shows its chemical composition: a steel with a high content of Cr, Mo and V.

The application of a design of experiments statistical technique aims to deliberately modify normal working conditions in order to produce changes in some of the studied responses. In industrial processes, it is common that a few factors are responsible for the majority of the variations in these responses. The factors analysed in a design of experiments may be quantitative and qualitative. Complete factorial experiments require a high number of experiments, which grow exponentially with regard to the number of factors to be studied. For example, when k factors are analysed, the number of experiments is 2^k^, where 2 is the number of values or levels that are applied to each factor. Fractional design of experiments allows a reduction in the number of complete factorial experiments. In fractional designs, there is a loss of information, which, in industrial practice, is not usually significant. The resolution of a design indicates the level of confounding that occurs in the estimation of its effects. In general, a resolution design R is that in which no effect of Q factors is confounded with another that contains fewer than R–Q factors. For example, if a design of experiments were considered with 6 factors and 8 experiments, its resolution would be III. That is to say, the main effects are confounded with the interactions of 2 factors. It may be confirmed that 3 (resolution) = 1 (main effects) + 2 (interactions of 2 factors). In this case, the design of experiments would be described as 2^5−2^_III_. Yates’ algorithm was applied to calculate main and interaction effects [24].

The effect of a factor on the variation of a response function is defined as a consequence of the variation of that factor. The effects of each separate factor are called the main effects. That is to say, the effect of a certain factor is defined as the change in a response function on variation of this factor between its level −1 and its level +1. The interactions between 2 factors are defined as the variation between the average effect of a factor with the other factor at its level −1, and the average effect of the same factor with the other factor at level +1. The interactions between various factors would be defined in a similar way.

**Figure 1:**
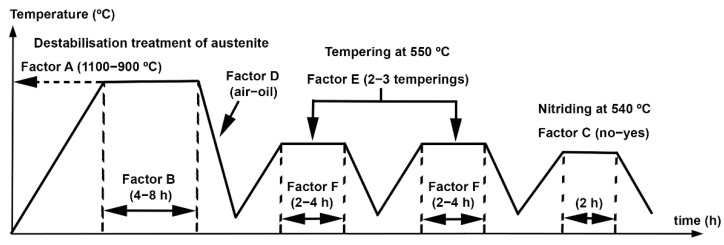
Diagram of the thermal process analysed through a design of experiments.

The experimental response is subject to random variation. This variation follows a normal law where its standard deviation reflects experimental error. The effects are linear combinations of responses so that, by applying the central limit theorem, they will follow a normal law. The distribution function associated with this will appear as a straight line if it is represented on the scale of a ‘normal probability paper’. The effects that are not significant will appear in line with this scale of representation (‘normal probability paper’). However, if one of the effects were significant, it would not appear in line with the non-significant effects, thus moving away from the straight line towards the extremes. This is due to the fact that these significant effects would not be included in the previously mentioned normal distribution. When significant factors appear associated with effects that move away from the straight line to the left and above it, it means that, if these factors are situated at their level −1, the response function with respect to its level +1 would increase. Similarly, when significant factors appear that move away from the straight line to the right and below it, this means that, if these factors are situated at their level +1, the response function would increase with respect to their level −1 [24]. Table 1 shows the factors and levels studied, and Table 2 shows the matrix of experiments. The column ‘restricted confounding patterns’ shows those second order interactions whose effects are confounded with each other and with the main factors. For example, in this case, they will be confounded in just one factor A and the interactions BD + CE. Factors A and B drive the destabilisation treatment of austenite. This destabilisation takes place through nucleation and growth kinetics. Therefore, this destabilization is driven by temperature and time. That is why two levels of temperature (1100 and 900 °C) and two levels of dwell time (4 and 8 h) are studied. Figure 1 shows a diagram of the thermal treatment process described in Table 2 and Table 3 in schematic form. Table 4 shows the parameters used in the plasma nitriding process.

The microstructures of the samples were analysed with a reflected light optical microscope NIKON Epiphot 200 (Nikon, Tokyo, Japan) and with a scanning electron microscope equipped with a system of energy-dispersive X-ray micro-analysis (EDX) model JEOL JSM-5600 (JEOL, Nieuw-Vennep, Netherlands). The percentages and types of co-existing crystalline phases were determined by X-ray diffraction (XRD, Baker Hughes, Celle, Germany), employing Cu as the emitting metal. The diffractometer used was a PANalyticalX’Pert Pro MPD. The Rietveld structural refinement method was used to identify the percentages of the crystalline phases. The wear tests were carried out in a pin-on-disc tribometer according to the Norm ASTM G99 and using Micro-Test MT/30/SCM/T (MicroTest, Madrid, Spain). Table 4 shows the main parameters of the process with which the plasma nitriding was carried out in experiments 5 to 8.

The analysed responses, by application of the design of experiments, were:

1. The following microstructural variables:
  ⚬ Weight percentage of tempered martensite
  ⚬ Weight percentage of retained austenite
  ⚬ Weight percentage of the carbides M_7_C_3_ and MC
2. Vickers hardness before nitriding. The load applied was of 300 N and the estimated hardness value in each experiment was of an average value obtained as from 12 hardness indentations.
3. Knoop hardness of the nitride layer in experiments 5 to 8. The load applied was of 0.5 N. The average estimated hardness value as from 12 hardness indentations carried out along the thickness of the nitrided layer.
4. The resistance to wear with a pin-on-disc test with a linear speed of 0.15 m/s and a load of 30 N. A ball of WC (with 6% Co) of 5 mm in diameter and a hardness of 1660 HV acted as the pin. The total distance travelled was 4 km.

The software used in the statistical analysis was Statgraphics Centurion XVI, version 16.1.03.

## 3. Results

**Figure 2:**
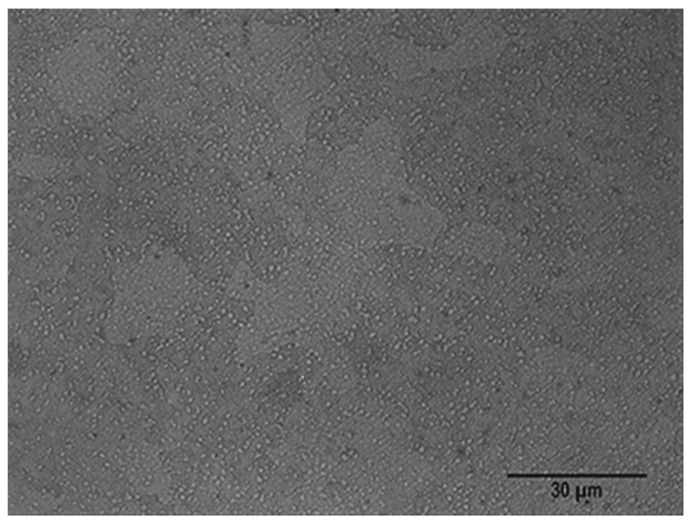
Microstructure of Vanadis 4 steel in an annealed state.

Figure 2 shows the microstructure of Vanadis 4 steel in an annealed state. The microstructure is made up of ferrite as the matrix constituent and a high density of carbides.

**Figure 3:**
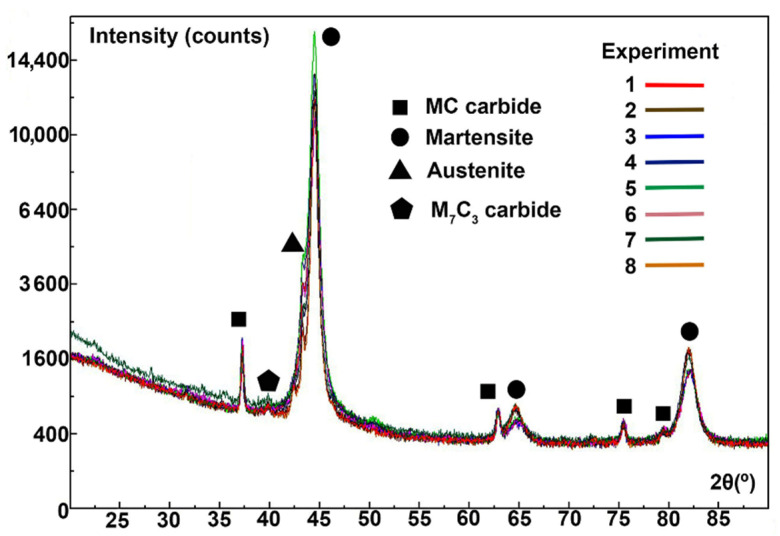
Diffractograms.

**Figure 4:**
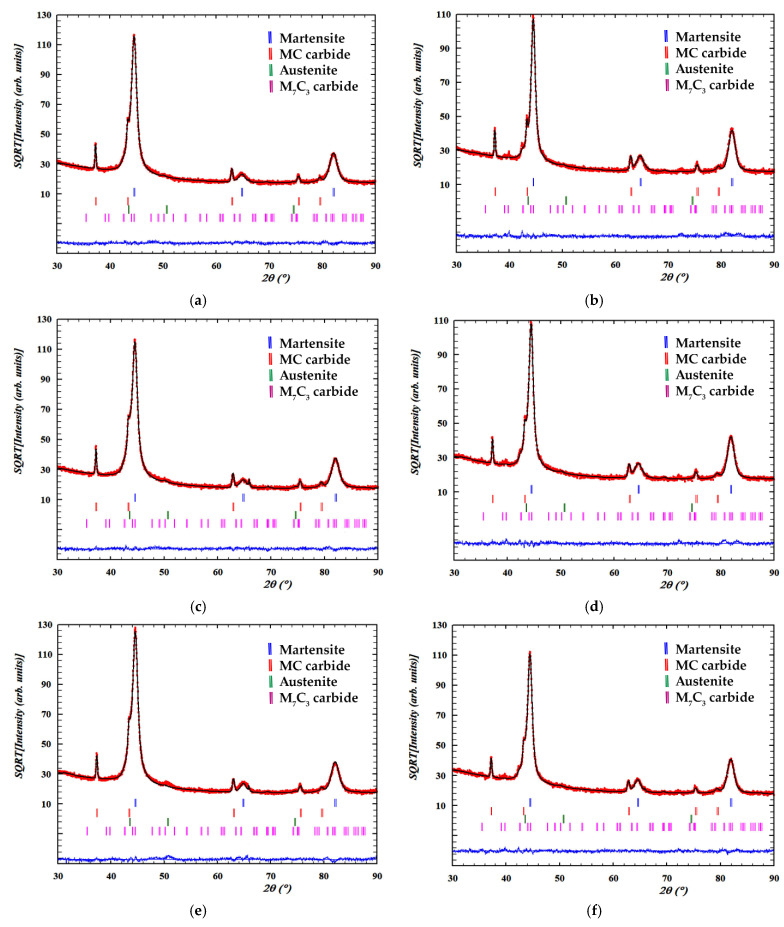

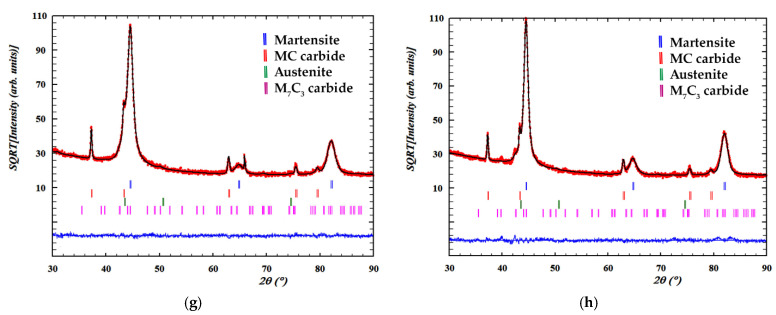
Overall fittings using the Rietveld method. Red crosses mark the observed intensities; the black line, the intensity calculated according to the Rietveld structural model; the bottom blue line, the difference between the two; the vertical bars, the positions of the reflections: martensite (blue); MC (red); austenite (green) and M_7_C_3_ (pink). The square root of intensity is represented on the abscissa axis. (**a**) Experiment 1; (**b**) Experiment 2; (**c**) Experiment 3; (**d**) Experiment 4; (**e**) Experiment 5; (**f**) Experiment 6; (**g**) Experiment 7; (**h**) Experiment 8.

Figure 3 shows the diffractograms obtained. The analysis was carried out after having performed all the heat treatments indicated in the matrix of experiments (Table 3). The Bragg peaks corresponding to martensite have been indexed to their reflections with Miller indices (110), (200) and (211). The Bragg peaks corresponding to austenite have been indexed to their reflections with the Miller index (111). Furthermore, other Bragg peaks can be appreciated in the irregular background produced by the fluorescence of the composition that has been identified with the structure of mixed carbides of type MC and M_7_C_3_. The individual profile of each Bragg peak was fitted using pseudo-Voigt functions. Table 5 provides the 2θ and intensity (I) values of the Bragg peaks that stood out the most. Figure 4 shows the overall fittings using the Rietveld method.

Table 6 shows the weight percentages and network parameters of the main crystalline phases detected by X-ray diffraction (XRD). It should be highlighted that, in all the experiments, the majority phase is tempered martensite, and the main carbides are of stoichiometry M_7_C_3_ (mixed carbide associated with Cr_7_C_3_) and stoichiometry MC (mixed carbide associated with VC). At the same time, it should be highlighted that, in only one of the experiments, the retained austenite slightly exceeds 10% in weight. This reflects the effectiveness of the treatments of destabilisation of austenite that have been proposed in this study.

Table 7 shows the average values obtained in each of the experiments. It also shows the effects corresponding to the restricted confounding pattern highlighted in the experiment matrix. In the first row of the columns of effects, the average value obtained with regard to the eight experiments is shown. For example, Table 7 points out that the average ferrite (tempered martensite) percentage obtained with regard to the eight experiments was 76.723 wt.%. Similarly, the average percentage of retained austenite obtained with regard to the eight experiments was 6.746 wt.%.

**Figure 5:**
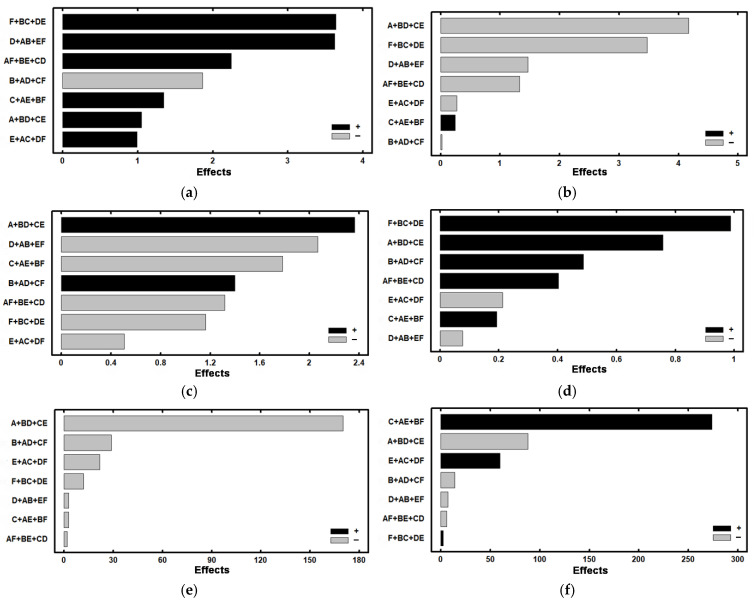

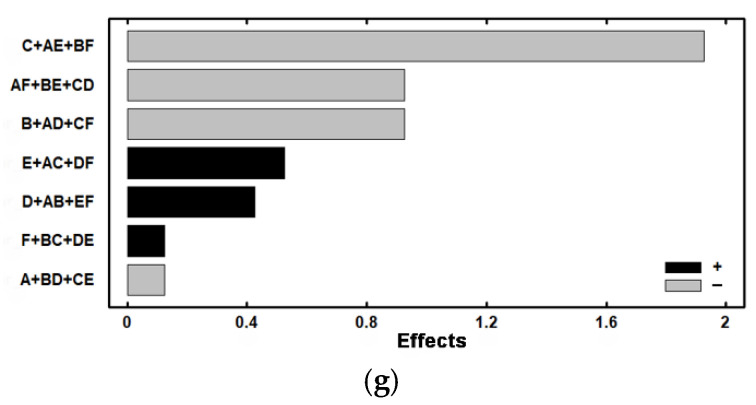
Representation of the effects in a Pareto diagram. On the ordinate axes, the main effects are shown, as well as second order effects, whose effect could be confounded with the effect of those main factors. On the abscissa axes, the main effects are shown, including the possible effect of second order interactions. (**a**) tempered martensite (wt.%); (**b**) retained austenite (wt.%); (**c**) carbides M_7_C_3_ (wt.%), associated with Cr; (**d**) carbides MC (wt.%), associated with V; (**e**) Vickers hardness before nitriding; (**f**) Vickers hardness after nitriding; (**g**) loss of mass due to wear (mg).

**Figure 6:**
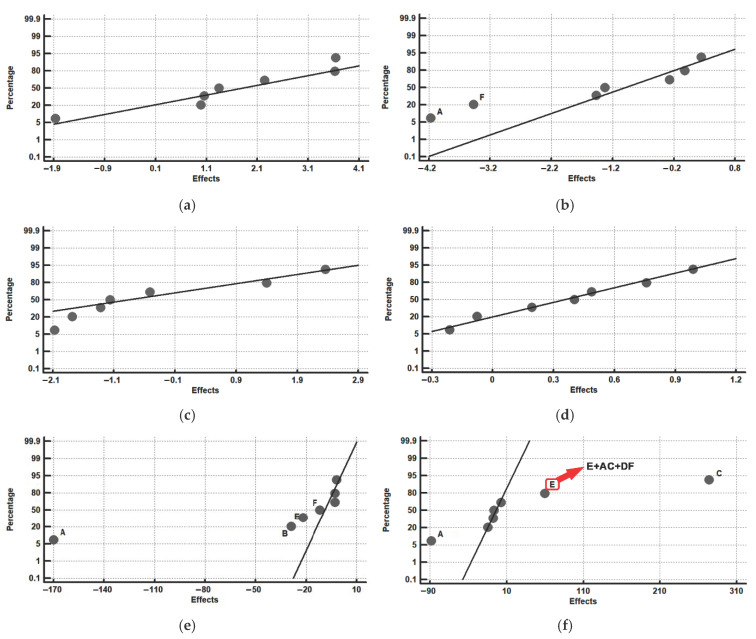

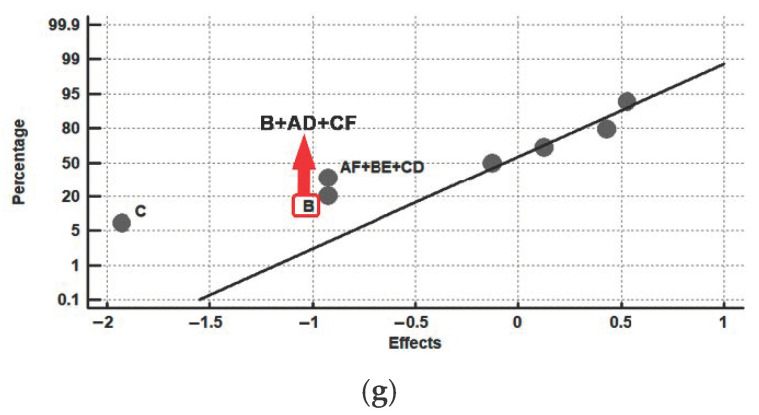
Representation of the effects on a normal probability paper. Those factors with a significant effect are shown. (**a**) tempered martensite; (**b**) retained austenite (wt.%); (**c**) carbides M_7_C_3_ (wt.%), associated with Cr; (**d**) carbides MC (wt.%), associated with V; (**e**) Vickers hardness before nitriding; (**f**) Vickers hardness after nitriding. In the case of the main factor E, second order interactions are shown, whose effect is confounded with the effect of factor E itself (interactions AC and DF); (**g**) loss of mass through wear (mg). In the case of the main factor B, second order interactions are shown, whose effect is confounded with the effect of factor B itself (interactions AD and CF).

Figure 5 shows the representation of the effects in Pareto diagrams, and Figure 6 shows the representation of the effects on a normal probability paper, highlighting those that present a significant effect in these responses. As well as the main significant effects, Figure 6 also includes second order interactions whose effects are confounded with the significant effect of factor E (Figure 6f) and the second order interactions whose effects are confounded with the significant effect of factor B (Figure 6g). The reason why, in these cases, the second order effects are highlighted is because, in Table 8 and Table 9, the effect of these second order interactions is analysed separately. 

**Figure 7:**
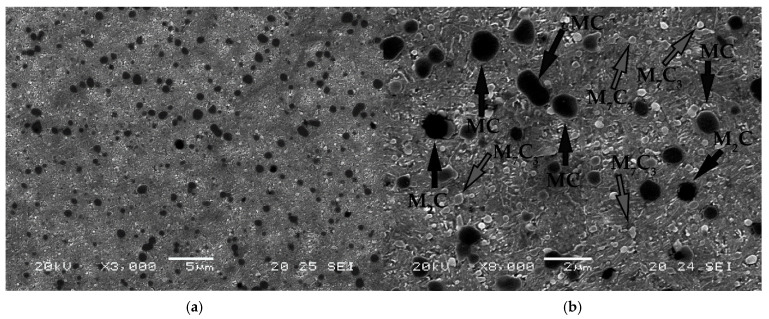

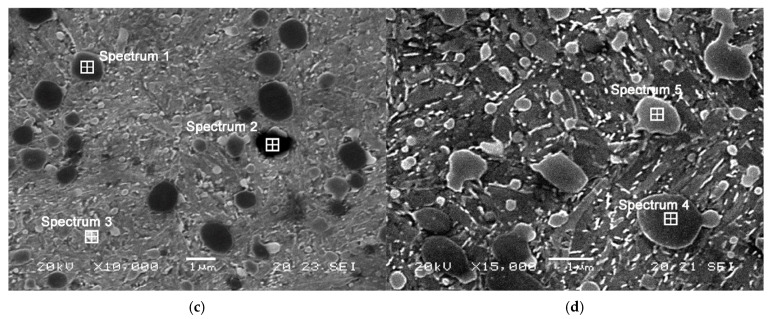
Representative microstructure after thermal treatments of quenching and tempering, before nitriding. (**a**–**c**) correspond to experiment 2 and (**d**) corresponds to experiment 4. In (**b**), the presence of M_2_C carbides, associated with Mo (colour black), are observed, as well as MC carbides, associated with V (colour dark grey), and carbides M_7_C_3_, associated with Cr (light grey).

Figure 7 shows the microstructure obtained after thermal treatments of quenching and tempering, before nitriding. Micrographs 7a to 7c correspond to experiment 2, while micrograph 4d corresponds to experiment 4. It appears that three types of carbides are differentiated. In Figure 7b, these carbides are highlighted by their different colouration. Table 8 shows the results obtained through the semi-quantitative analysis of these three types of carbides. It must be pointed out that the presence of mixed carbides associated with Mo_2_C (M_2_C), apparently in very small quantities, has been detected. These were not detected in the DRX analysis. These carbides are those that have a black colouration in the micrograph in Figure 7b. Together with these carbides, a much more abundant quantity of carbides MC (associated with vanadium) is observed. These appear with a lighter colour and a smaller size with respect to the former in Figure 7b.

**Figure 8:**
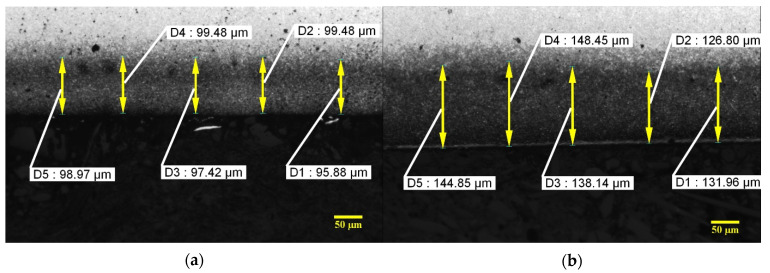

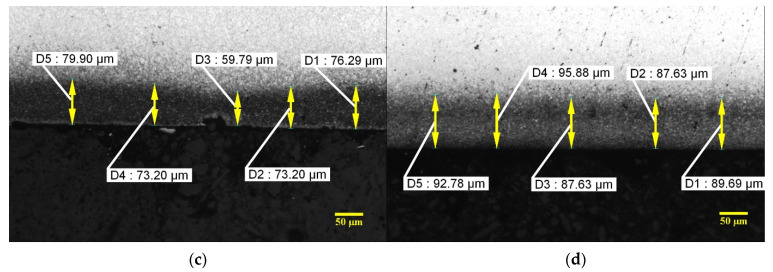
Thicknesses of the nitrided layer. (**a**) Experiment 5; (**b**) Experiment 6; (**c**) Experiment 7; (**d**) Experiment 8.

Figure 8 shows the thicknesses of the nitrided layer. These thicknesses are of around 100 microns, reaching a maximum value of around 150 microns and minimum values of around 60 to 70 microns.

**Figure 9:**
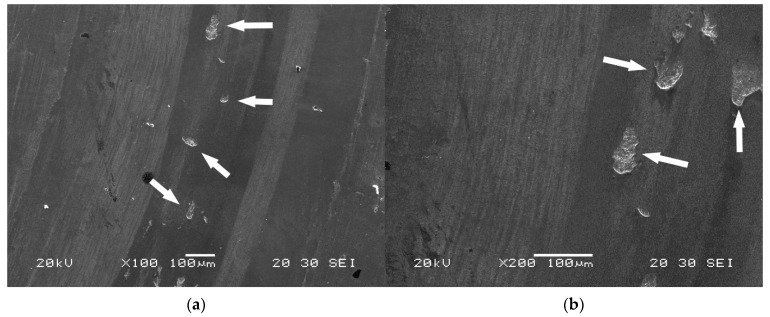

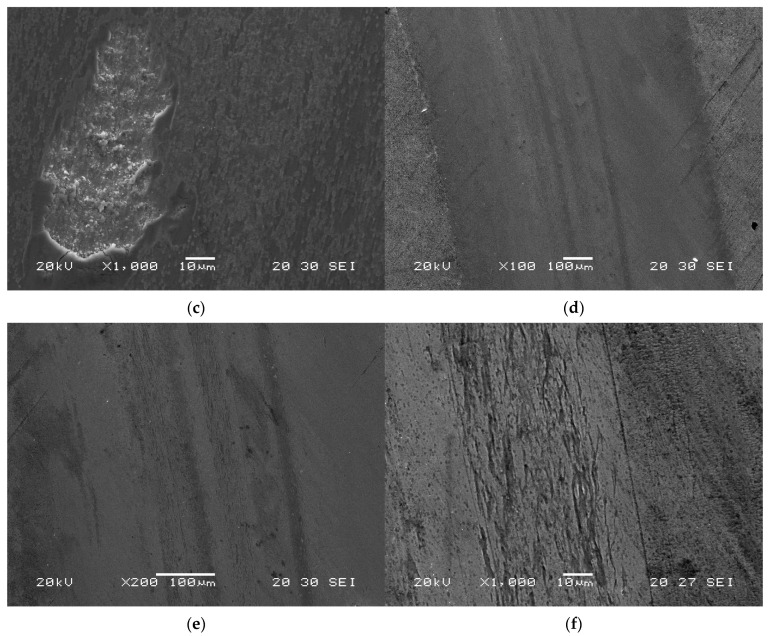
Surface of wear in the “pin-on-disc” test. (**a**–**c**) correspond to experiment 1 (without nitriding). It is shown that the breaking off of solid particles is produced by a mechanism of abrasive wear of ‘two bodies’; (**d**–**f**) correspond to experiment 6 (with nitriding). A largely adhesive mechanism of wear is shown.

Wear is produced by the loss of material in the interphase of two bodies subjected to a relative movement with the action of a load. In this case, the two bodies in contact were the pin (ball of WC) and the disc (the test materials). The load applied was 30 N. Figure 9 shows the interphase of wear corresponding to experiment 1, without nitriding treatment, and experiment 6, with nitriding treatment. It can be observed that, in the case where the surface is not nitrided, the breaking off of solid particles may occur and these remain trapped between the contact surfaces, thus producing a mechanism of abrasive wear. However, in the case of a nitrided surface, the mechanism of wear appears to be fully adhesive. Nitriding produces a large increase in hardness, which favours greater resistance to wear, just as has been shown in the previously analysed results.

## 4. Discussion

Figure 6a shows that none of the analysed factors has a significant effect on the percentage of tempered martensite. However, if the corresponding Pareto diagram is taken into consideration, Figure 5a, it is shown that the factors that have a greater effect on this percentage are F (tempering time) and D (means of quenching). This means that, to increase the percentage of this phase, it would be recommended to situate both factors at their levels +1: oil quenching and 4 h of tempering at 550 °C.

If Figure 5b and Figure 6b are taken into consideration, it is concluded that factors A (temperature of austenisation) and F (tempering time) have a significant effect on the percentage of retained austenite. Therefore, if we wish to reduce the retained austenite, we should situate both factors at their level +1, that is to say, temperatures of destabilisation at 900 °C and tempering times of 4 h. It may be deduced that a temperature of destabilisation of the austenite of 1100 °C would be excessive for favouring the precipitation of secondary carbides. Thus, if this temperature is situated at 900 °C, a greater precipitation of secondary carbides would be produced. Moreover, the Ms temperature would be raised with respect to that which would be obtained if the destabilisation temperature was 1100 °C. On the other hand, long temperings (4 h) at 550 °C favour a second destabilisation of the austenite and its transformation into martensite.

Figure 6c,d shows that none of the studied factors have a significant effect on the percentage of carbides M_7_C_3_ and MC. However, if the two factors with a greater effect in the respective Pareto diagrams are considered, Figure 5c,d, it may be observed that: (1) to increase the percentage of M_7_C_3_ carbides, factor A should be situated at its level +1 (destabilisation temperature of 900 °C) and factor D at its level −1 (air quenching), and (2) to increase the percentage of MC carbides, the F and A factors should be situated at their +1 levels (temperings of 4 h at 550 °C and destabilisation temperature at 900 °C). These results confirm that the most effective temperature for destabilisation of the austenite would be 900 °C, that air quenching favours the precipitation of type M_7_C_3_ carbides in the interval of temperature between 600 °C and 400 °C [25,26] and that, during prolonged temperings at 550 °C, the precipitation of V is favoured. This would have remained dissolved in the austenite after quenching. With prolonged times of tempering at 550 °C, this would give time for the unprecipitated V, which occupies substitutional positions in the tempered martensite, to migrate by diffusion towards the dislocations of the ferrite and to react with C to form mixed MC carbides of nanometric size [17,27].

Figure 6e shows that the factors that have a significant effect on hardness, before nitriding treatment, are factors A (temperature of destabilisation), B (time of permanence at the temperature of destabilisation), E (number of temperings) and F (time of tempering). Therefore, in order to increase hardness, all of these factors should be situated at their levels −1. That is to say: destabilisation of the austenite at 1100 °C for 4 h, with a maximum of two temperings and short tempering times (2 h). The destabilisation temperature of the austenite at 1100 °C is excessive for complete destabilisation of the austenite with short dwell times (4 h). Thus, during quenching, the martensite formed would be more alloyed and would present greater hardness. At the same time, short tempering times would maintain a certain tetragonality of the tempered martensite, and, finally, greater hardness [16]. With respect to this, it must be pointed out that, before tempering, the martensite contains Cr, V and Mo in substitutional solid solution. At high tempering temperatures, as is the case of 550 °C, the cementite precipitated during low tempering temperatures has been dissolved by decomposition of its integrating atoms (Fe and C). The atoms Cr, V and Mo at 550 °C have the energy to slowly diffuse towards crystalline defects, react with C and precipitate. The M_7_C_3_ carbides tend to increase in size if the duration of tempering is long. However, the MC carbides are much more resistant to coalescence, maintaining their nanometric size for longer and a fine dispersion of the tempered martensite [17].

Figure 6f shows hardness after the nitriding treatment. As well as the nitriding (factor C) itself being significant, the significant effect of factor E (number of temperings) and also factor A (destabilisation temperature) must be highlighted. In order to increase hardness, both factors should be situated at their respective levels +1 (three temperings) and −1 (destabilisation at 1100 °C). However, the significant effect of factor E requires a more detailed analysis. In this effect, the effect of the interactions AC and DF is included, as well as factor E. That is to say, in the effect associated with the main factor E, the effects associated with the interactions AC and DF are also included. Table 9 shows the analysis of their effects. It may be seen that it is the interaction AC that has a significant effect and not factor E. Therefore, if factors C (nitriding) and A (destabilisation temperature) are situated simultaneously at their respective levels +1 and −1, a complementary increase in hardness is produced.

Figure 6g shows the loss of mass by wear. Factor C (nitriding) and effect B (dwell time at destabilisation temperature) both have a significant effect. Wear would be increased without the nitriding treatment and, apparently, with short destabilisation times (4 h). It seems logical to assume that, when a nitriding treatment is not carried out, the rate of wear increases. However, the effect associated with the main factor B includes the effect of the interactions AD and CF. Here, factor C (nitriding) appears again. That is to say, the effects caused by interactions AD and CF are also included in the effects of factor B. Because of this, it seems reasonable to analyse the effect of the interaction CF in greater detail. In Table 10, the independent effects of these interactions are analysed. It is concluded that the most significant effect is the interaction CF, increasing the rate of wear at their levels (C = −1 and F = +1). That is to say, resistance to wear decreases if, as well as not carrying out the nitriding treatment (factor C at its level −1), tempering times are increased to 4 h (factor F at its level +1). This reduces the hardness of tempered martensite. In contrast, it can be seen that lower loss of mass is obtained by situating both factors at their levels +1, that is to say, by carrying out the nitriding treatment with a previous tempering of 4 h. Prolonged temperings at 550 °C favour the precipitation of VC of nanometric size. The V occupies substitutional positions, and, apart from high temperatures for dissipation, it needs enough time to dissipate and reach precipitation by heterogeneous nucleation in the dislocations and network defects of the ferrite. On the other hand, the significant effect of interactions AF, BE and CD may be observed. Table 11 shows the results of their separate analysis. It is concluded that the interaction with a significant effect is interaction CD, in such a way that resistance to wear would increase if both factors were simultaneously at their level +1. That is to say, simultaneous oil quenching and nitriding treatment would be favourable for a decrease in the rate of wear.

These results show that greater resistance to wear is achieved when the material is subjected to a nitriding treatment. This is more effective when prior oil quenching with double temperings of 550 °C for 4 h is carried out. Oil quenching with temperings of 4 h favours optimum microstructure against wear: a high-volume fraction of martensite with a reduction in retained austenite, together with the presence of vanadium carbides of nanometric size, precipitated during temperings of 4 h.

## 5. Conclusions

Vanadis 4 steel is a steel for tools processed by powder metallurgy. Through the application of a design of experiments with six factors and eight experiments, the parameters of thermal processing have been analysed. Among these are those related to the destabilisation of the austenite, the cooling method in quenching, tempering and the carrying out of an ionic nitriding treatment. The main conclusions are:

1. The most effective destabilisation temperature would be 900 °C. At this temperature, a greater precipitation of secondary carbides is produced, thus raising the Ms temperature and favouring a reduction in retained austenite with values of less than 8% in weight. To complement this, if a double tempering of 4 h at 550 °C is carried out, this percentage falls to 3%.
2. Nitriding significantly increases hardness, reaching values of 900 HV. This hardness is boosted if the destabilisation treatment is carried out at 1100 °C. This temperature is excessive for complete destabilisation of the austenite; therefore, the martensite formed during quenching presents greater hardness. At the same time, short tempering times maintain a certain tetragonality in the tempered martensite, thus favouring an increase in hardness.
3. Resistance to wear increases notably with the nitriding treatment. To complement this, oil quenching and tempering times of 4 h are recommended. Resistance to wear increases with tempering at 500 °C for four hours. This favours the precipitation of nanometric MC carbides, associated with V.

## Figures and Tables

**Table 1:** Chemical composition (wt.%).

C	Si	Mn	Cr	Mo	V
1.4	0.4	0.5	4.7	3.5	3.7

**Table 2:** Factors and levels.

Factors		Levels	
Code	Description of Factors	Level −1	Level +1
A	Destabilisation temperature (°C)	1100	900
B	Dwell time at destabilisation temperature (h)	4	8
C	Nitriding	No	Yes
D	Quench cooling medium	air	oil
E	Nº of temperings at 550 °C	2	3
F	Time of tempering (h)	2	4

**Table 3:** Matrix of experiments (2^5−2^_III_).

No.	A	B	C	D	E	F	Restricted Confounding Pattern
1	−1	−1	−1	+1	+1	+1	A + BD + CE B + AD + CF C + AE + BF D + AB + EF E + AC + DF F + BC + DE AF + BE + CD
2	+1	−1	−1	−1	−1	+1	
3	−1	+1	−1	−1	+1	−1	
4	+1	+1	−1	+1	−1	−1	
5	−1	−1	+1	+1	−1	−1	
6	+1	−1	+1	−1	+1	−1	
7	−1	+1	+1	−1	−1	+1	
8	+1	+1	+1	+1	+1	+1	

**Table 4:** Parameters used in the plasma nitriding process.

Gas Mixture	70%N_2_ + 30%H_2_
Gas flux (cm^3^/min)	500
Temperature (°C)	540
Pressure (Pa)	400
Time (min)	120
Output voltage (V)	500

**Table 5:** 2θ (deg.) and I (counts) values obtained from the fitting of the Bragg peaks.

**Experiment**	**Martensite**						**Austenite**	
	**2θ**	**I**	**2θ**	**I**	**2θ**	**I**	**2θ**	**I**
1	44.442	8907	64.710	109	81.994	399	43.676	1220
2	44.436	7901	64.613	192	81.936	600	43.778	722
3	44.426	8669	64.699	98	81.934	405	43.689	1489
4	44.408	7171	64.488	243	81.829	911	43.773	1093
5	44.427	10,189	64.761	120	82.025	405	43.850	1150
6	44.467	8350	64.550	192	81.911	524	43.802	1313
7	44.516	7158	64.816	101	82.049	403	43.824	425
8	44.456	8152	64.640	210	81.950	584	43.681	332
**Experiment**	**MC carbides**							
	**2θ**	**I**	**2θ**	**I**	**2θ**	**I**	**2θ**	**I**
1	37.213	1246	62.858	203	75.396	111	79.454	41
2	37.208	1122	62.833	194	75.373	113	79.306	26
3	37.176	1391	62.797	222	75.336	127	79.290	32
4	37.180	786	62.777	213	75.296	128	79.248	37
5	37.206	1122	43.393	2297	75.415	107	79.425	23
6	37.249	877	62.830	159	75.349	96	79.179	26
7	37.262	1329	62.892	236	75.435	123	79.484	34
8	37.214	988	62.841	184	75.377	98	79.325	37
**Experiment**	**M_7_C_3_ carbides**							
	**2θ**				**I**			
1	43.037				361			
2	42.377				236			
3	42.380				148			
4	42.376				134			
5	42.423				141			
6	42.531				186			
7	42.481				144			
8	42.362				180			

**Table 6:** Microstructural parameters, weight distributions of the precipitated phases and volume of the unit cell.

No.	Rietveld Fitting	Phases	a (Å)	c (Å)	wt. %	Vol. (Å^3^)
1	R_wp_ = 7.73 R_exp_ = 5.95 Chi^2^ = 1.69	Martensite	2.87514		79.46 ± 1.98	23.767 ± 0.004
		Austenite	3.60122		6.78 ± 0.64	46.704 ± 0.014
		M_7_C_3_	7.04759	4.53305	9.40 ± 1.71	194.986 ± 0.73
		MC	4.17342		4.36 ± 0.55	72.690 ± 0.016
2	R_wp_ = 10.1 R_exp_ = 6.99 Chi^2^ = 2.09	Martensite	2.87696		78.14 ± 2.26	23.812 ± 0.003
		Austenite	3.60122		3.02 ± 0.68	46.704 ± 0.014
		M_7_C_3_	7.04759	4.53305	13.02 ± 2.10	194.986 ± 0.73
		MC	4.17785		5.81 ± 0.70	72.817 ± 0.015
3	R_wp_ = 7.52 R_exp_ = 5.79 Chi^2^ = 1.68	Martensite	2.87675		72.58 ± 1.90	23.807 ± 0.004
		Austenite	3.60122		10.37 ± 0.72	46.704 ± 0.014
		M_7_C_3_	7.04759	4.53305	12.71 ± 1.88	194.986 ± 0.73
		MC	4.17789		4.34 ± 0.54	72.924 ± 0.014
4	R_wp_ = 9.42 R_exp_ = 6.68 Chi^2^ = 1.99	Martensite	2.88094		74.02 ± 2.17	23.911 ± 0.004
		Austenite	3.60122		6.33 ± 0.73	46.704 ± 0.014
		M_7_C_3_	7.04759	4.53305	14.83 ± 2.17	194.986 ± 0.73
		MC	4.18092		4.83 ± 0.66	73.083 ± 0.019
5	R_wp_ = 8.23 R_exp_ = 5.43 Chi^2^ = 2.3	Martensite	2.87462		78.42 ± 1.83	23.754 ± 0.004
		Austenite	3.60122		9.44 ± 0.65	46.704 ± 0.014
		M_7_C_3_	7.04759	4.53305	7.97 ± 1.53	194.986 ± 0.73
		MC	4.17055		4.18 ± 0.55	72.541 ± 0.015
6	R_wp_ = 8.87 R_exp_ = 6.68 Chi^2^ = 1.76	Martensite	2.88061		74.60 ± 2.23	23.903 ± 0.004
		Austenite	3.60122		7.79 ± 0.77	46.704 ± 0.014
		M_7_C_3_	7.04759	4.53305	13.21 ± 2.18	194.986 ± 0.73
		MC	4.18027		4.40 ± 0.69	73.049 ± 0.018
7	R_wp_ = 8.62 R_exp_ = 6.18 Chi^2^ = 1.94	Martensite	2.87563		74.33 ± 2.06	23.779 ± 0.003
		Austenite	3.60122		8.74 ± 0.73	46.704 ± 0.014
		M_7_C_3_	7.04759	4.53305	11.59 ± 1.97	194.986 ± 0.73
		MC	4.17578		5.33 ± 0.65	72.814 ± 0.011
8	R_wp_ = 9.96 R_exp_ = 7.01 Chi^2^ = 2.02	Martensite	2.87724		82.24 ± 2.36	23.819 ± 0.004
		Austenite	3.60122		1.50 ± 0.67	46.704 ± 0.014
		M_7_C_3_	7.04759	4.53305	10.06 ± 2.02	194.986 ± 0.73
		MC	4.17644		6.20 ± 0.75	72.848 ± 0.017

**Table 7:** Average values and effects obtained for the analysed responses: ferrite (tempered martensite) and austenite, carbides; hardness (before and after nitriding) and pin-on-disc test.

**Experiment**	**Ferrite**		**Austenite**		**Effect**
	**(wt.%)**	**Effect**	**(wt.%)**	**Effect**	
1	79.46	76.723	6.78	6.746	Average
2	78.14	1.052	3.02	−4.172	A + BC + CE
3	72.58	−1.862	10.37	−0.022	B + AD + CF
4	74.02	1.347	6.33	0.242	C + AE + BF
5	78.42	3.622	9.44	−1.467	D + AB + EF
6	74.6	0.992	7.79	−0.272	E + AC + DF
7	74.33	3.637	8.74	−3.472	F + BC + DE
8	82.24	2.242	1.50	−1.327	AF + BE + CD
**Experiment**	**Cr_7_C_3_**		**VC**		**Effect**
	**(wt.%)**	**Effect**	**(wt.%)**	**Effect**	
1	9.40	11.598	4.36	4.931	Average
2	13.02	2.362	5.81	0.757	A + BC + CE
3	12.71	1.397	4.34	0.487	B + AD + CF
4	14.83	−1.782	4.83	0.192	C + AE + BF
5	7.97	−2.067	4.18	−0.077	D + AB + EF
6	13.21	−0.507	4.40	−0.212	E + AC + DF
7	11.59	−1.162	5.33	0.987	F + BC + DE
8	10.06	−1.317	6.20	0.402	AF + BE + CD
**Experiment**	**Before Nitriding**		**After Nitriding**		**Effect**
	**HV (300 N)**	**Effect**	**HV (300 N/0.5 N)**	**Effect**	
1	732	648.5	732	786.9	Average
2	585	−170.0	585	−88.2	A + BC + CE
3	716	−29.0	716	−14.2	B + AD + CF
4	567	−3.0	567	273.7	C + AE + BF
5	761	−3.0	937 ^1^	−7.2	D + AB + EF
6	574	−22.0	922 ^1^	59.7	E + AC + DF
7	725	−12.0	939 ^1^	2.7	F + BC + DE
8	528	−2.0	897 ^1^	−6.2	AF + BE + CD
**Experiment**	**Wear (Pin on Disk Test)**				**Effect**
	**mg**		**Effect**		
1	4.2		1.7		Average
2	2.2		−0.1		A + BC + CE
3	1.8		−0.9		B + AD + CF
4	2.5		−1.9		C + AE + BF
5	0.7		0.4		D + AB + EF
6	1.6		0.5		E + AC + DF
7	0.4		0.1		F + BC + DE
8	0.3		−0.9		AF + BE + CD

^1^ Hardness taken in the nitrided layer. The Knoop test was used with an applied load of 0.5 N.

**Table 8:** Semi-quantitative analysis of the carbides highlighted in Figure 4. Micro-analysis by energy dispersive X-rays (EDX). (atomic %).

Spectrum	%V	%Cr	%Fe	%Mo	Mixed Carbide
1	20.67	2.89	25.84	4.44	MC
2	10.62	4.23	35.27	16.81	M_2_C
3	0.91	4.74	61.94	1.25	M_7_C3
4	16.84	4.31	30.62	4.38	MC
5	0.75	5.51	63.11	0.94	M_7_C3

**Table 9:** Analysis of the effect of interactions AC and DF on hardness after nitriding treatment. (↓) represents the column (down), while (→) represents the row (to the right).

A(↓) × C(→)	−1	+1	D(↓) × F(→)	−1	+1
−1	724	938	−1	819	762
+1	576	909	+1	752	814

**Table 10:** Analysis of the effect of the interactions AD and CF on wear after nitriding treatment.

A(↓) × D(→)	−1	+1	C(↓) × F(→)	−1	+1
−1	1.1	2.4	−1	2.15	3.2
+1	1.9	1.4	+1	1.15	**0.3**

**Table 11:** Analysis of the effect of the interactions AF, BE and CD on wear.

A(↓) × F(→)	−1	+1	B(↓) × E(→)	−1	+1	C(↓) × D(→)	−1	+1
−1	1.2	2.3	−1	1.4	2.9	−1	2.0	**3.3**
+1	2.0	1.2	+1	1.4	1.0	+1	1.0	**0.5**

## Data Availability

Data are contained within the article.
